# Analysis of Sn-Bi Solders: X-ray Micro Computed Tomography Imaging and Microstructure Characterization in Relation to Properties and Liquid Phase Healing Potential

**DOI:** 10.3390/ma14010153

**Published:** 2020-12-31

**Authors:** Georg Siroky, Elke Kraker, Jördis Rosc, Dietmar Kieslinger, Roland Brunner, Sybrand van der Zwaag, Ernst Kozeschnik, Werner Ecker

**Affiliations:** 1 Materials Center Leoben Forschung GmbH (MCL), Roseggerstraße 12, 8700 Leoben, Austria; elke.kraker@mcl.at (E.K.); joerdis.rosc@mcl.at (J.R.); roland.brunner@mcl.at (R.B.); werner.ecker@mcl.at (W.E.); 2 ZKW Elektronik GmbH, Samuel Morse-Straße 18, 2700 Wiener Neustadt, Austria; dietmar.kieslinger@zkw-electronic.at; 3 Faculty of Aerospace Engineering, TU Delft, Kluyverweg 1, 2629 Delft, The Netherlands; s.vanderzwaag-1@tudelft.nl; 4 Institute of Materials Science and Technology, TU Wien, Getreidemarkt 9, 1060 Wien, Austria; ernst.kozeschnik@tuwien.ac.at

**Keywords:** solder, X-ray, computed tomography, morphology, anisotropy, FEM, healing, semi-solid, elastic, RVE

## Abstract

This work provides an analysis of X-ray micro computed tomography data of Sn-xBi solders with x = 20, 30, 35, 47, 58 wt.% Bi. The eutectic thickness, fraction of eutectic and primary phase are analyzed. Furthermore, the 3D data is evaluated by means of morphology parameters, such as, shape complexity, flatness, elongation and mean intercept length tensor. The investigated alloys are categorized in three groups based on their morphology, which are described as “complex dominant”, “complex- equiaxed” and “mixed”. The mechanical behavior of Sn-Bi alloys in the semi-solid configuration and the correlation with microstructural parameters are discussed. A varying degree of geometric anisotropy of the investigated alloys is found through the mean intercept length tensor. Representative volume element models for finite element simulations (RVE-FEM) are created from tomography data of each alloy to analyze a correlation of geometric and elastic anisotropy. The simulations reveal an elastic isotropic behavior due to the small difference of elastic constants of primary and eutectic phase. A discussion of properties in the semi-solid state and liquid phase healing is provided.

## 1. Introduction

Efforts to reduce the environmental impact of microelectronic products led to the development of lead-free solder alloys with varying content of Ag, Bi, In, Cu, Ga and Zn among other alloying elements [1,2,3,4,5,6]. The effort to reduce the environmental impact of Pb containing solders led to several alloys of varying Sn, Ag and Cu content [7]. Recently, low melting point solders gained interest in the research community, where the Sn-Bi system is a promising candidate for soldering of temperature-sensitive components [2]. Studies investigated Sn-Bi alloys with respect to alloying elements [8,9,10], thermal parameters [11], magnetic stirring [12] and directional solidification [13,14]. The effect of In addition in Sn-Bi alloys on melting point and mechanical properties was shown by Wu et al. [8] and microstructural effects due to Cu and Ag additions were investigated by Silva et al. [9]. A strengthening effect of Ag addition to Sn-Bi alloys was reported by Ren et al. [10]. Elastic properties and their temperature-dependence were investigated by means of pulse echo overlap method [12], ultrasonic transmission [15,16] and nano-indentation [3,17]. Despite the thorough mechanical characterization, a 3D microstructural analysis of Sn-Bi alloys based on tomographic data is missing in literature. 

Micro X-ray computed tomography (µ-XCT) imaging offers a non-destructive spatial analysis of materials and parts [18]. It was applied on solder materials to investigate several microstructural features such reflow porosities [19,20,21] or phase morphology [22,23,24,25,26]. The size distribution of spherical reflow porosities in solder joints was reported by Jiang et al. [19] and Rauer et al. [20]. Shi et al. [21] investigated the deformation of reflow porosities in fatigue experiments with µ-XCT imaging and finite element simulations. A combined approach of µ-XCT imaging and focused ion beam (FIB) tomography was reported by Yazzie et al. [22] and highlighted the local microstructure and morphology of Sn-rich solders. The size distribution and morphology of intermetallic phases in Sn-rich solders was reported by Kaira et al. [24] and the primary phase dendrites in Sn-Pb solder were assessed with µ-XCT imaging by Mertens et al. [25]. A three-dimensional morphometric characterization of Sn-Ag-Cu solder microstructures was reported by Maleki at al. [26]. The microstructural evolution of flip-chip solder joints under electromigration were investigated by [27] using in-situ 3D laminography and finite element simulations. X-ray tomography data of solders was used for numerical modelling of thermal and mechanical properties, where Michael et al. [28] used tomography data to numerically investigate the thermal resistance of solder joints with respect to reflow porosities. The elasto-plastic response of Sn-Ag-Cu solders after aging was studied by Maleki et al. [26,29] and highlighted the possibility to compute the stress-strain response from tomography data. Besides these reported studies on thermal and mechanical behavior, other properties also depend on the microstructural morphology, such as semi-solid deformation [30,31,32], hot tearing [33,34] or liquid phase healing [35,36]. These properties have not been reported in literature for Sn-Bi alloys and it is the aim of the present work to provide the essential parameters to enable such studies. 

The volumetric phase fraction and local grain or phase thickness are commonly evaluated parameters and can be determined from 2D- [37] and 3D measurements [25]. In addition, the mean intercept length (MIL) parameter is used to describe the geometric anisotropy [38]. It quantifies the number of phase boundaries along randomly oriented trajectories through the volume of interest (VOI). The degree of anisotropy (*DA*) can be derived from the eigenvalues of the MIL tensor and provides a scalar measure with *DA* = 0 representing an isotropic and *DA* = 1 a perfectly aligned anisotropic microstructure [38]. A new morphology categorization was reported by Fang et al. [39], which is based on the shape complexity parameter [40,41], elongation and flatness [42,43]. They determined shape classes based on these three morphology parameters and distinguished among spherical, equiaxed, rod, sheet and complex shaped domains [39]. This enables the quantification of morphologic similarities among alloys and provides the basis for generic microstructure creation.

This study provides an analysis of several microstructural descriptors from µ-XCT images. Five samples of varying Bi content (20 wt.%, 30 wt.%, 35 wt.%, 47 wt.% and 58 wt.% Bi) and a microstructure representative of that obtained during industrial reflow processing are prepared. The reconstructed tomography data is segmented into Sn-rich primary- and Bi-rich eutectic phase and microstructural parameters are evaluated from the segmented tomography data, such as, eutectic phase fractions, mean intercept length of phase boundaries, volume to surface ratio, complexity parameter, flatness and elongation. The mechanical behavior of Sn-Bi alloys in the semi-solid state is discussed using the extracted morphology parameters and literature data. Representative volume element models for finite element simulations (RVE-FE) are derived from the µ-XCT data for finite element simulations and the correlation between geometric and elastic anisotropy is investigated. The work ends with a morphology-based prediction of which alloy composition is most likely to yield the best liquid phase-assisted self-healing behavior. 

## 2. Materials and Methods

### 2.1. Sample Preparation

**Figure 1:**
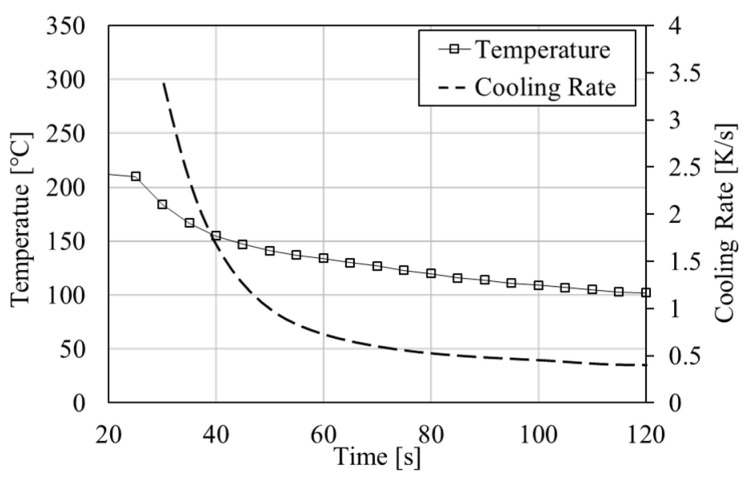
Temperature (solid squares) and cooling rate (dashed) during sample casting Sn-20 wt.% Bi sample.

Alloys of composition Sn- 20, 30, 35, 47 and 58 wt.% Bi were prepared on an induction heat plate and held at 250 °C for 60 min for homogenization. Cylindrical samples of dimension ø 3 mm × 10 mm were cast in a silicon mold and solidified under air cooling. The temperature in the silicon mold was measured using a contact thermometer. The temperature and cooling rates in the mold are illustrated in Figure 1. The average cooling rate in the initial phase of solidification was 1.5 K/s. The solidus temperature of $T_{s}$ = 138 °C was reached after 55 s. Cylindrical pins of ø 0.5 mm × 1 mm were machined from the as-cast samples on a lathe under oil cooling. The pins were glued to cylindrical glass rods for positioning in the µ-XCT setup. Samples were prepared for SEM (scanning electron microscopy) imaging by mechanical grinding and polishing for comparison with measurements from µ-XCT data. The homogeneity of the sample microstructure was assessed by measuring the eutectic phase fraction from SEM cross-sections, where sample dependencies were insignificant. 

Images of sample cross-sections were obtained on a SEM (FEI Philips XL30, Philips, Amsterdam, The Netherlands) in electron back scatter mode (BSE) and with 20kV acceleration voltage. 

### 2.2. Imaging and Segmentation

The samples were scanned on a lab-scale µ-XCT device (GE nanotom m, General Electric, Boston, MA, USA) with an isotropic voxel size of about 1.8 µm. The acceleration voltage was set to 116 kV and the X-ray tube current was 100 µA. These settings were chosen to allow transmission in the samples and to minimize image artefacts. No pre-filters were applied on the X-ray tube and the VOI of scanned samples had the dimensions of approximately 400 × 400 × 1050 µm^3^. 

The Sn-rich primary phase and Bi-rich eutectic phase were segmented with the commercial image analysis software Avizo 2019.1 (www.thermofischer.com). Prior to segmentation, the signal-to-noise ratio was enhanced by applying a median filter with a kernel of 3 × 3 × 3 voxels, which improves the detectability of phase boundaries. The segmentation of dendrites was achieved using the top-hat transform algorithm. This includes two steps, first, a closing operation on the grey value image and second, the selection of a threshold value to detect the dark primary phase regions in the data. This approach reduces noise, improves the detectability of structures on the length scale of several voxels and reduces non-uniformities caused by beam-hardening artefacts. 

**Figure 2:**
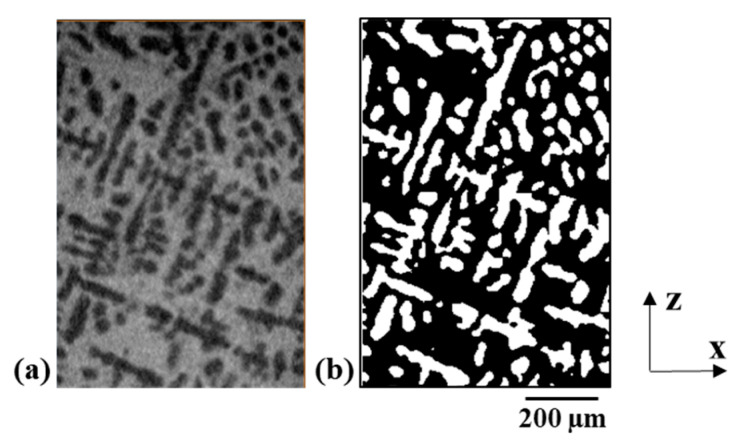
Representative image obtained in the x-z plane from the 3D data for the Sn 58 wt.% Bi alloy. (**a**) Grey value image displays the primary phase (dark grey) and eutectic phase (light grey). (**b**) Corresponding segmented primary phase (white).

**Figure A1:**
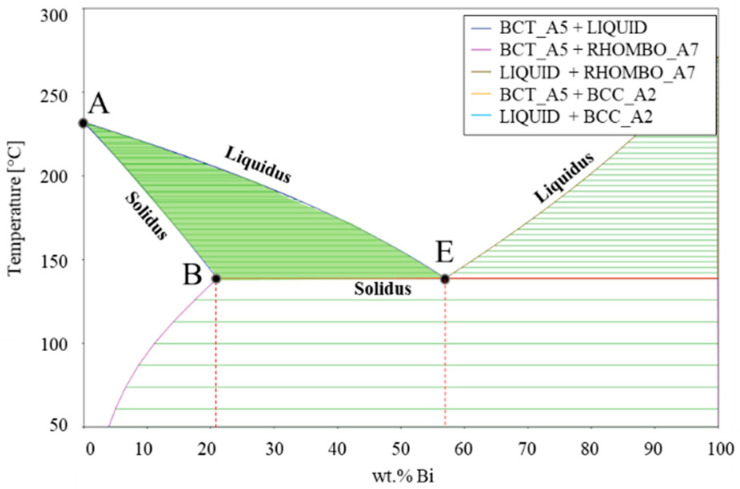
Sn-Bi binary phase diagram.

The segmentation delivered a binary data set which consists of the primary phase and the surrounding eutectic phase. A representative grey value image after image acquisition and the segmented primary phase are illustrated in Figure 2a,b, respectively. The comparison of grey value and segmented image in Figure 2a,b shows that morphological features, such as, dendrites or phase boundaries are well represented. The segmented data is compared with scanning electron microscopy (SEM) images to verify the eutectic thickness. Furthermore, phase fractions from µ-XCT measurements are compared with CALPHAD (Calculations of Phase Diagrams) calculations using the software package MatCalc 6.03 (http://matcalc.at) with the thermodynamic database COST 531 [44], to quantify the agreement of theoretical and measured phase fractions. A phase diagram of the binary Sn-Bi system is provided in Figure A1 (Appendix A).

### 2.3. Microstructural Parameter

The segmented images were analyzed using the open-source package ImageJ 1.53c [45,46,47]. Connected domains were analyzed and labelled with a random color lookup table for rendering. Several microstructural quantities are extracted from the segmented 3D data, such as, eutectic volume fraction, eutectic surface fraction, eutectic thickness, MIL tensor, degree of anisotropy (*DA*), volume of eutectic ($V_{e}$), total VOI volume ($V_{\mathrm{total}})$ and surface area of eutectic ($A_{e}$). The eutectic volume fraction, $\phi_{e}$, is calculated with

(1) $$\phi_{e}=\frac{V_{e}}{V_{\mathrm{total}}}$$

and compared with CALPHAD calculations. The eutectic surface fraction, $\psi_{e}$, is obtained through

(2) $$\psi_{e}=\frac{A_{e}}{V_{\mathrm{total}}}$$

The eutectic thickness, $t_{e}$, is defined according to Hildebrand and Rüegsegger [47] as the largest diameter of a sphere completely inside the eutectic phase, which is written as

(3) $$t_{e}\left(x_{e}\right)=2\cdot \max\left(\left\{r|x_{e}\in \mathrm{sph}\left(x,r\right)\subseteq \Omega ,x\in \Omega \right\}\right)$$

where $\Omega \subset R^{3}$ is the set of all points within the eutectic structure and $x_{e}\in \Omega$ is an arbitrary point in the eutectic structure. The expression sph(x,r) define the points inside a sphere with center x and radius r. A possible VOI size-dependence of several extracted parameters, such as, $\phi_{e}$, $A_{e},\;t_{e}$ or *DA* is provided in the Appendix B in Figure A3, Figure A4, Figure A5 and Figure A6 respectively. A morphology analysis based on the shape complexity, elongation and flatness was performed following the classifications found to be useful in describing the healing of damage during creep loading of steels [39]. The shape complexity parameter, $\Omega_{3}$, is calculated as

(4) $$\Omega_{3}=\frac{A}{\pi^{\frac{1}{3}}{\left(6V\right)}^{\frac{2}{3}}}$$

with *A* and *V* being the surface area and volume of the segmented domain, respectively. Furthermore, the elongation and flatness are evaluated based on the moment of inertia, $I_{j}$, of an equivalent ellipsoid. The semi-axes of the ellipsoid, $a_{j}$, are calculated from the eigenvalues of the inertia tensor, I**,** as

(5) $$a_{j}=\;\sqrt{\frac{5\left(tr\left(I\right)-2I_{j}\right)}{2V}}$$

The elongation is calculated accordingly with

(6) $$E=\frac{2a_{1}}{a_{2}+a_{3}}$$

and the flatness with

(7) $$F=\frac{a_{2}}{a_{3}}$$

Five shape categories are defined according to their combination of $\Omega_{3}$, E and F, which is summarized in Table 1. A discussion on the correlation of shape and morphology parameters is given in [39]. 

An ellipsoid fit of the MIL tensor is computed [46] and the *DA* is calculated from the associated eigenvalues, $\lambda_{i}$, with

(8) $$DA=1-\frac{\min\left(\lambda_{i}\right)}{\max\left(\lambda_{i}\right)}$$

### 2.4. RVE-FEM Model

Voxel-based RVE-FE models were created from µ-XCT data to investigate the agreement with elastic mixing rules and a possible elastic anisotropy. The segmented binary images were converted into the mhd format and a voxel mesh was generated using the software package medtool 4.4 (www.dr-pahr.at/medtool). A coarsening factor of 3 was applied on the 3D data, leading to an average voxel size of 6 μm, where a convergence analysis with a coarsening factor ranging from 3 to 5 was performed. Cuboid models with *x*, *y* and *z* dimensions of 356 µm × 356 µm × 1029 µm and kinematic periodic boundary conditions (PBC) were created in the commercial software package ABAQUS 6.19 (www.3ds.com). The microstructure was approximated with 596,991 elements of type C3D8. Material parameters for elastic properties of primary and eutectic phase were taken from literature [17] and values are provided in Table 2. The Young’s moduli of microstructures with 20, 30, 47 and 58 wt.% Bi were evaluated numerically under uniaxial deformation in x-, y- and z- direction. For comparison with the *DA* parameter, the elastic anisotropy is evaluated from simulations as

(9) $$DA_{\mathrm{el}}=1-\frac{\min(E_{i})}{\max(E_{i})}$$

with $\min(E_{i})$ and $\max(E_{i})$ representing the minimal and maximal Young’s modulus, respectively. 

The linear and inverse rules of mixture were evaluated according to [48] for comparison with RVE-FE results. 

## 3. Results

### 3.1. Tomographic Rendering

**Figure 3:**
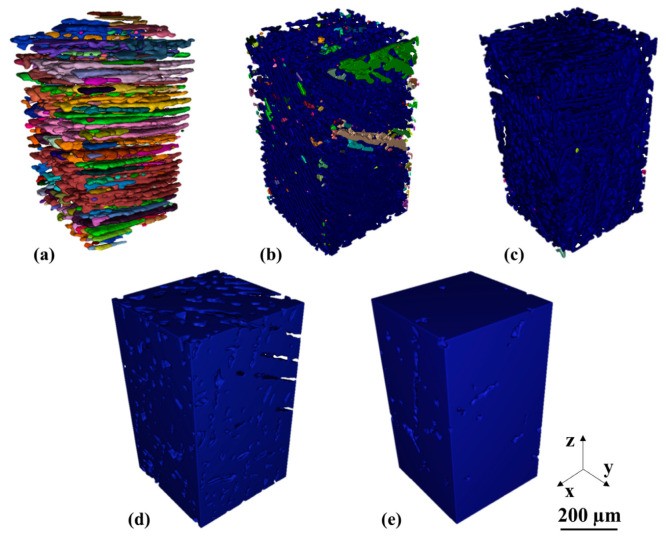
Segmented eutectic phase obtained from µ-XCT. Color code: Each color represents a single connected phase. (**a**) Sn-20 wt.% Bi (**b**) Sn-30 wt.% Bi (**c**) Sn-35 wt.% Bi (**d**) Sn-47 wt.% Bi (**e**) Sn-58 wt.% Bi.

Figure 3 shows a rendering of the segmented eutectic phase from µ-XCT data of five alloy compositions, where the color indicates connected domains. The Sn-20 wt.% Bi sample in Figure 3a reveals the highest fragmentation with isolated islands of eutectic phase. The Sn-30 wt.% Bi sample in Figure 3b exhibits fewer disconnected regions, where a large connected eutectic domain is indicated in blue. The samples with 35, 47 and 58 wt.% Bi are illustrated in Figure 3c,d,f, respectively. These latter compositions show a connected network of eutectic phase across the entire VOI.

**Figure 4:**
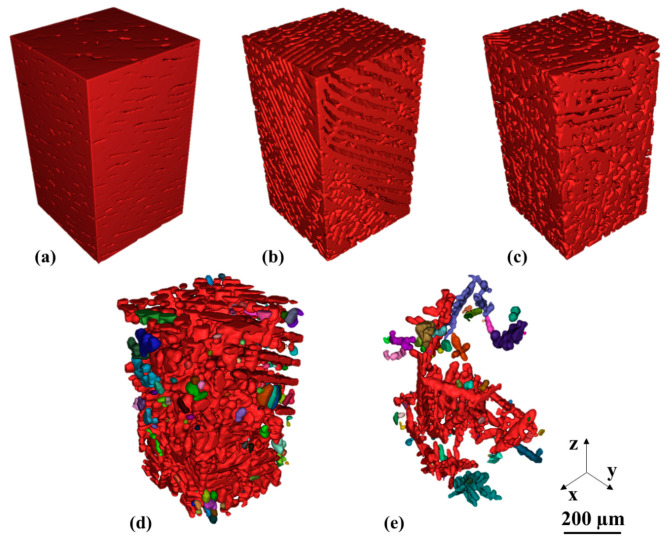
Segmented primary phase obtained from µ-XCT. Color code: Each color represents a single connected phase. (**a**) Sn-20 wt.% Bi (**b**) Sn-30 wt.% Bi (**c**) Sn-35 wt.% Bi (**d**) Sn-47 wt.% Bi (**e**) Sn-58 wt.% Bi.

Complementary images of the primary phase are shown in Figure 4. A network is indicated by red colored domains in Figure 4a–c. A comparison among Figure 3c and Figure 4c of the Sn-35 wt.% Bi alloy shows an interpenetrating network of the primary and eutectic phases with a low fragmentation of domains. The Sn-47 wt.% Bi and Sn-58 wt.% Bi reveal separated dendritic structures of primary phase in Figure 4c,d, respectively. Despite the lower primary phase fraction in the Sn-58 wt.% Bi alloy, a connectivity of dendritic structures is visible. Furthermore, the primary and secondary dendrites are captured in Figure 4e.

### 3.2. Volume Fractions

**Figure 5:**
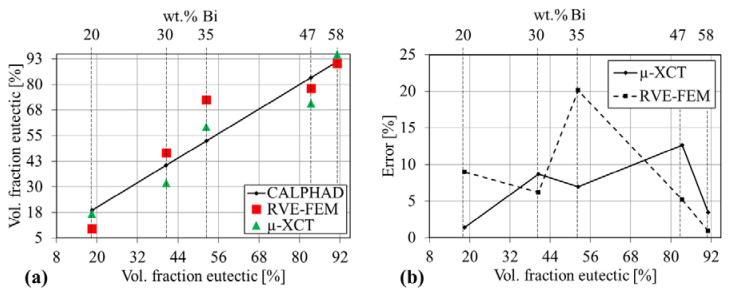
Eutectic volume fraction obtained from CALPHAD calculations, image analysis of µ-XCT data and the RVE-FE model. (**a**) eutectic volume fraction over composition (**b**) relative error of µ-XCT data and RVE-FE model based on equilibrium CALPHAD calculations over composition.

**Figure A2:**
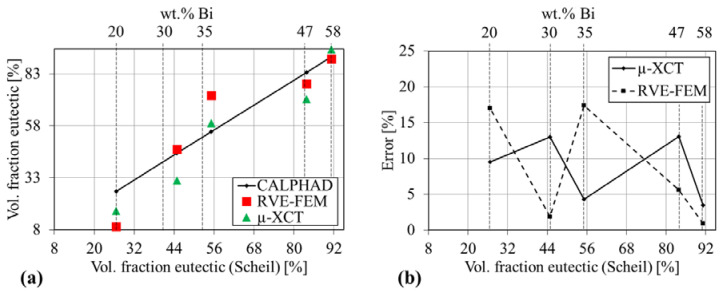
Eutectic volume fraction from CALPHAD calculations, µ-XCT imaging and the RVE-FE model (**a**) eutectic volume fraction over composition (**b**) relative error of µ -XCT data and RVE-FE model based on Scheil CALPHAD calculations over composition.

**Figure A3:**
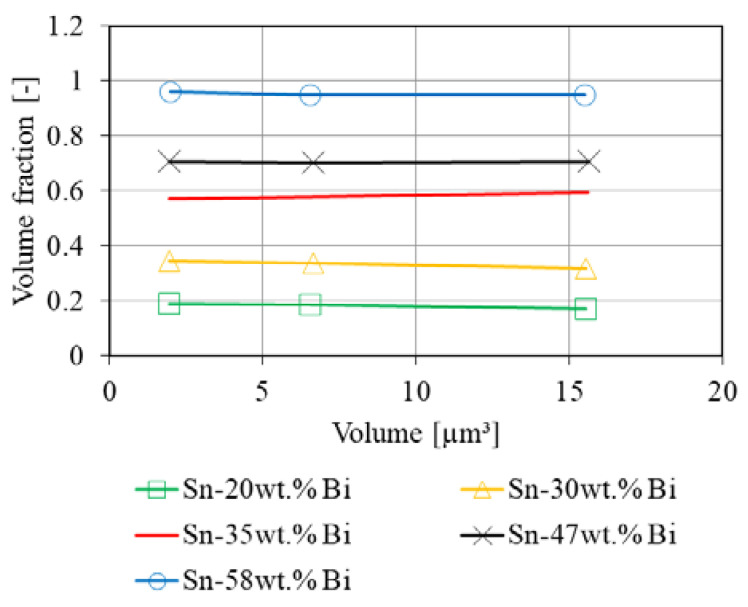
Volume fraction over variation of VOI and alloy composition.

The eutectic volume fraction is extracted from the µ-XCT data and illustrated in Figure 5, where a comparison with CALPHAD calculations and the generated RVE-FE models is given. Figure 5a shows a correlation between the equilibrium eutectic fraction (black solid) with µ-XCT (green triangles) and RVE-FE (red squares) values. The µ-XCT data captures the increasing eutectic fraction, with slight deviations from the predicted CALPHAD phase fractions (black solid line). The deviations seem random, with compositions 20, 30 and 47 wt.% Bi underestimating and 35 and 58 wt.% overestimating the theoretical eutectic fraction. Due to the image coarsening of the RVE-FE models, these trends are further amplified. Figure 5b shows the error compared with CALPHAD calculations of µ-XCT and RVE-FE data. The deviation of the µ-XCT data remains below 10% for most alloys except the Sn-47 wt.% Bi composition. The same holds for the RVE-FE data, whereas the Sn-35 wt.% Bi alloy deviates considerable with 20%. This originates from an unfavorable approximation due to the voxel-mesh. A refinement of the voxel-mesh would lead to a better representation of the eutectic fractions. However, a limit of about $5\times 10^{6}$ elements prevents a further refinement and the Sn-35 wt.% Bi alloy was omitted from simulations. A comparison with Scheil calculations showed similar results with deviations from the theoretical values of about 10%. Results of the Scheil analysis are given in the Figure A2 and a variation of VOI and its effect on the volume fraction is given in Figure A3.

### 3.3. Eutectic Thickness

**Figure 6:**
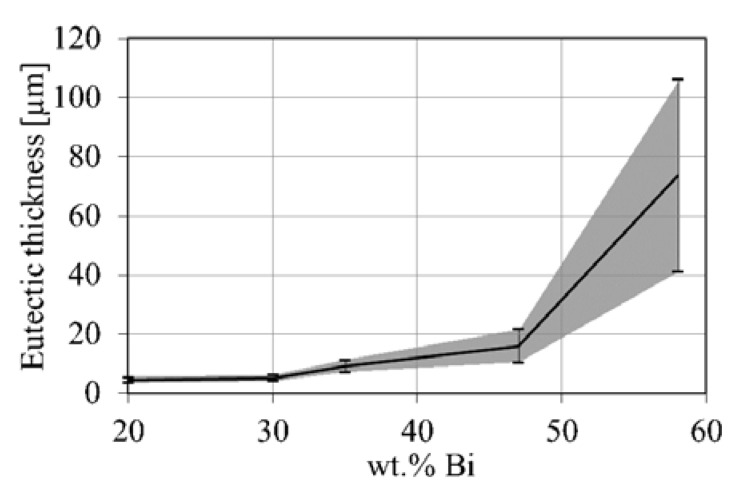
Eutectic phase thickness obtained from µ-XCT measurements as a function of Bi content. Error bars indicate the standard deviation of eutectic thickness.

The eutectic thickness determined from µ-XCT data is shown in Figure 6. The eutectic thickness ranges from 3.6 µm for the Sn-20 wt.% Bi alloy to 73 µm for the Sn-58 wt.% Bi alloy. The data indicates a linearly increasing eutectic thickness among alloys with 20, 30, 35 and 47 wt.% Bi content. The Sn-58 wt.% Bi alloy shows the largest mean eutectic thickness and highest scatter of the measured values. Furthermore, a steep increase of mean eutectic thickness is observed from 47 to 58 wt.% Bi. The large scatter might arise from the dendritic microstructure and the large eutectic network observed in Figure 4e.

### 3.4. Morgphology Parameters

**Figure 7:**
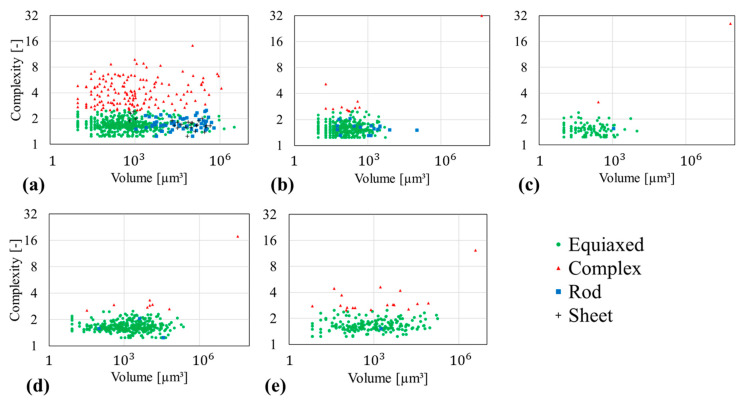
Calculated complexity parameter based on the segmented µ-XCT data as a function of the domain volume for Sn-Bi alloys. Morphology classes are equiaxed (green), complex (red), rod (blue), sheet (grey). (**a**) Sn-20 wt.% Bi (**b**) Sn-30 wt.% Bi (**c**) Sn-35 wt.% Bi (**d**) Sn-47 wt.% Bi (**e**) Sn-58 wt.% Bi.

**Figure 8:**
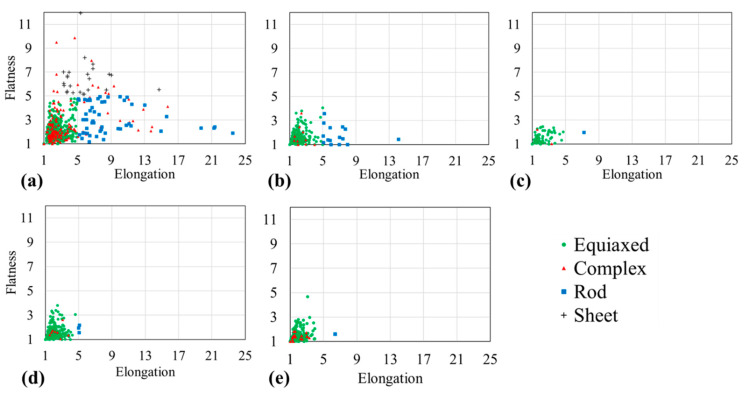
Flatness over elongation for Sn-Bi alloys based on the segmented µ-XCT data. Morphology classes are equiaxed (green), complex (red), rod (blue), sheet (grey). (**a**) Sn-20 wt.% Bi (**b**) Sn-30 wt.% Bi (**c**) Sn-35 wt.% Bi (**d**) Sn-47 wt.% Bi (**e**) Sn-58 wt.% Bi.

The morphology parameters $\Omega_{3},\;E,\;F$ of each Sn-Bi alloy are given in Figure 7 and Figure 8. The analysis shows that their microstructure is composed of equiaxed-, complex-, rod- and sheet-shaped domains. The shape complexity, $\Omega_{3}$, over volume is presented in Figure 7. The illustrated data points represent each segmented sub-domain and its corresponding shape class. The Sn-20 wt.% Bi alloy in Figure 7a is composed of equiaxed, complex, rod and sheet domains, where sheet and rod-like structures tend towards larger sub-volumes of $10^{3}$–$10^{6}\;{\mu m}^{3}$. The equiaxed and complex-shaped domains are spread across the total volume range. The other alloys of 30, 35, 47 and 58 wt.% Bi in Figure 7b–e, respectively, reveal similar complexity values. Each alloy contains a complex-shaped outlaying data point with a volume of around $10^{7}\;{\mu m}^{3}$, which is the connected network of primary and eutectic phase. The majority of the data points is equiaxed-shaped with a volume of 10–$10^{5}\;{\mu m}^{3}$. 

Flatness over elongation in Figure 8 gives further morphological information on the microstructure. The Sn-20 wt.% Bi alloy is illustrated in Figure 8a and shows the maximum range of elongation (1–25) and flatness (1–11) among the analyzed alloys. The rod-shaped structures have a maximum flatness *F* of 5 and an elongation *E* ranging from 5 to 25. Most data points are equiaxed and complex-shaped, where low values of *E* and *F* indicate uniformly-shaped structures. The 30, 35, 47 and 58 wt.% Bi alloys in Figure 8b–e reveal high similarity in the *E*-*F* space, where, except for a few rod-shaped structures, most domains are equiaxed- and complex-shaped. Furthermore, the Sn-35 wt.% Bi alloy in Figure 8c reveals the most isotropic geometry with $E_{\max}=3$ and $F_{\max}=7.2$ among the analyzed compositions. A large spread in *E*–*F* space is associated with a strong geometric orientation. Low and similar values among *E* and *F* characterize isotropic microstructures.

### 3.5. Geometric Anisotropy

**Figure 9:**
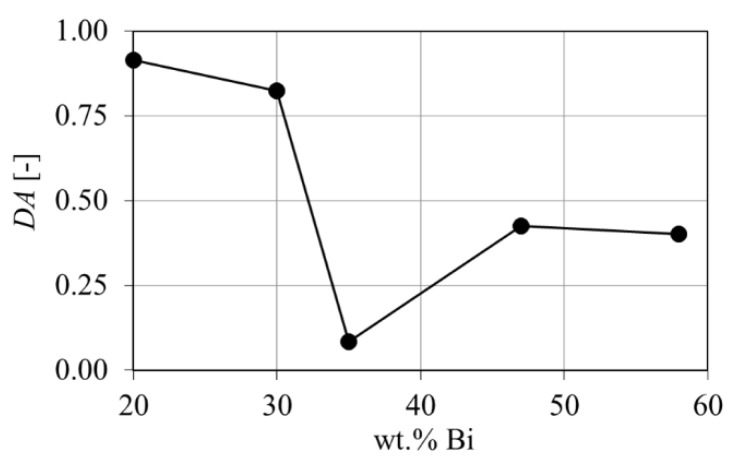
Calculated degree of anisotropy from µ-XCT measurements over composition of Sn-Bi alloys.

The geometric orientation is analyzed using the MIL tensor and *DA* parameter, where *DA* = 0 is spatial isotropy and *DA* = 1 fully anisotropic [46]. Eigenvalues and eigenvectors of the ellipsoid on the MIL tensor are given in Appendix C in Table A1 and the respective ellipsoids are provided in Appendix C in Figure A7. Figure 9 shows the *DA* over composition, where the Sn-20 wt.% Bi alloy reveals the highest degree of anisotropy. This is in agreement with the wide span of *E* and *F* in Figure 8a, which indicated a flat and elongated microstructure. Furthermore, the Sn-35 wt.% Bi gives the lowest value of *DA* = 0.058, which is reflected by a low spread of *E* and *F* values in Figure 8c. A VOI size variation with respect to *DA* is given in Figure A6 and showed an insignificant variation with respect to VOI dimensions.

### 3.6. Computed Elastic Response

**Figure 10:**
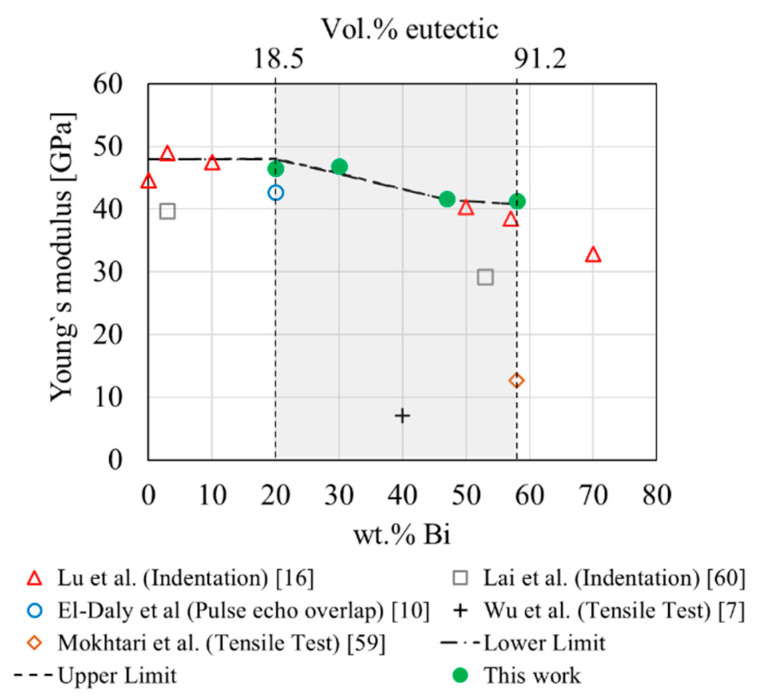
RVE-FE results, linear/inverse mixing rule and literature values of Young’s modulus.

In Figure 10, the Young’s moduli calculated through RVE-FE models are shown (green, solid-dot). The analytical linear and inverse mixing rules were evaluated (dashed) for comparison, where graphs of linear and inverse mixing rule appear congruent due to the low difference in Young‘s moduli of primary and eutectic phase. Furthermore, values for Young’s moduli reported in literature are shown as a benchmark for computed results. The RVE-FE model shows good agreement with results from the analytical mixing rules. The RVE-FE calculations in Figure 10 are also in good agreement with Young’s moduli reported by Lu et al. [17] through nano-indentation and values by El-Daly [12] through pulsed echo overlap (PEO). Values of Wu et al. [8], Mokhtari et al. [49] and Lai et al. [50] show considerable deviation from the RVE-FE values arising from the specific measurement methods used, where tensile tests in particular underestimate the elastic properties.

## 4. Discussion

### 4.1. Eutectic Surface Fraction

**Figure 11:**
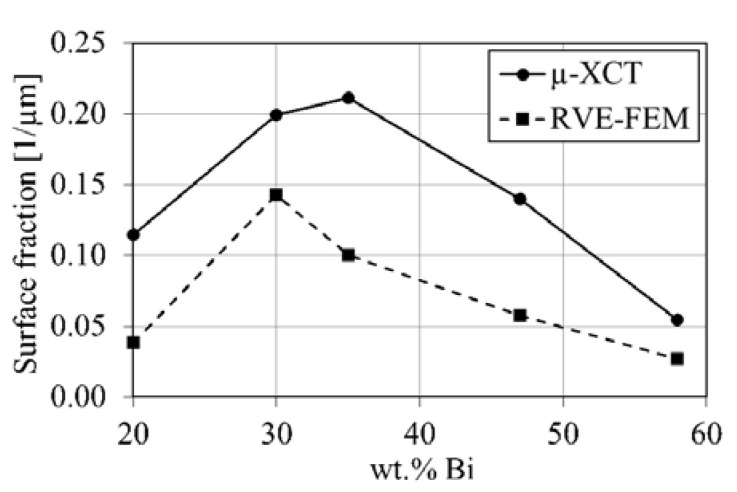
Eutectic surface fraction over composition from µ-XCT (solid) measurements and RVE-FE (dashed) model.

**Figure A4:**
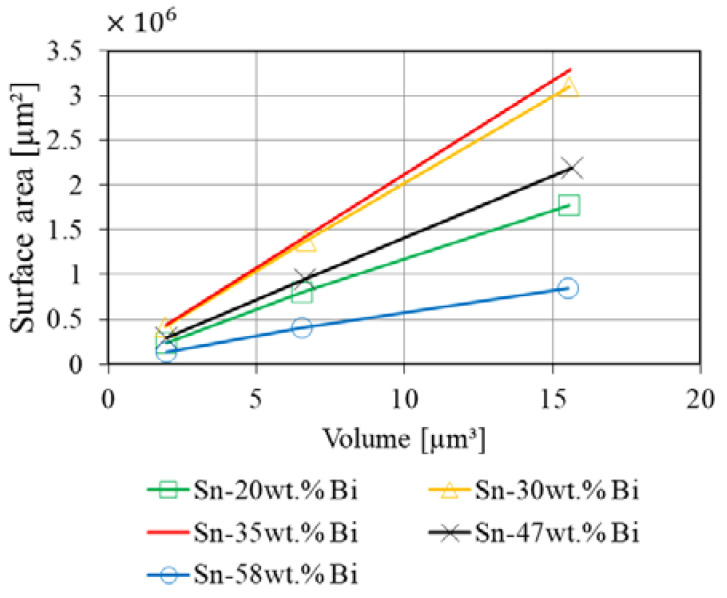
Eutectic surface area over variation of VOI and alloy composition.

The eutectic surface fraction according to Equation (2) for the µ-XCT data and RVE-FE models is given in Figure 11. The µ-XCT data shows a maximum surface fraction for the Sn-35 wt.% Bi alloy, which corresponds to the interpenetrating network of eutectic- and primary phase observed in Figure 3 and Figure 4. Furthermore, it reveals that Sn-Bi alloys with around 50% eutectic phase fraction create the maximum number of phase boundaries. The RVE-FE model in Figure 11 properly reflects the tomography data. Yet, the surface fraction in the RVE-FE model is underestimated on average by roughly 50%, which arises from the voxel approximation. Therefore, the model in its presented form has limitations with respect to properties that require a quantitative representation of phase boundaries. Nevertheless, the general trend is fulfilled for alloys with 20, 30, 47 and 58 wt.% Bi. The phase boundary surface area with respect to VOI size is given in Figure A4.

### 4.2. Eutectic Thickness: SEM & µ-XCT

**Figure 12:**
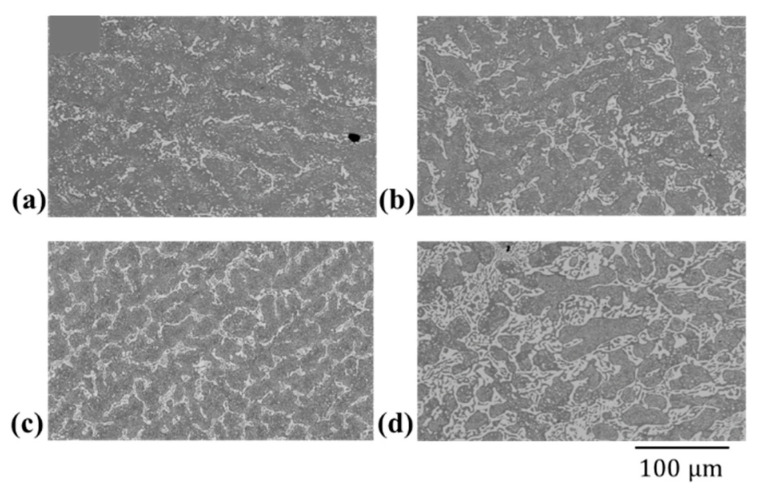
SEM cross-sections of Sn-Bi alloys (**a**) Sn-20 wt.% Bi (**b**) Sn-30 wt.% Bi (**c**) Sn-35 wt.% Bi (**d**) Sn-47 wt.% Bi. Scale bar with 100 µm applies to all images.

**Figure 13:**
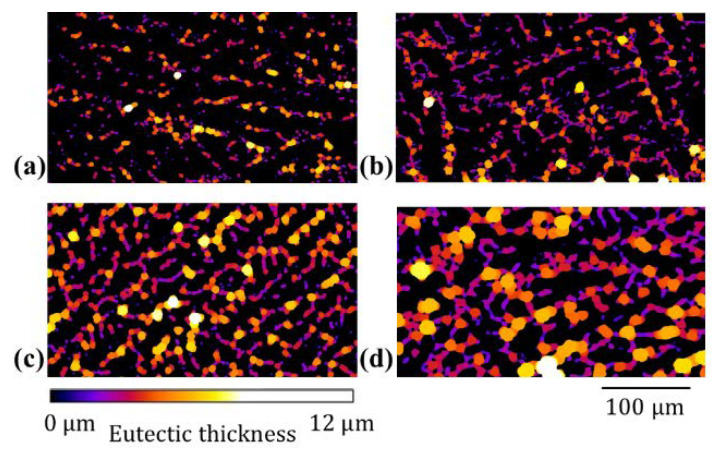
Local eutectic thickness map of Sn-Bi alloys (**a**) Sn-20 wt.% Bi (**b**) Sn-30 wt.% Bi (**c**) Sn-35 wt.% Bi (**d**) Sn-47 wt.% Bi. The color spectrum defines the eutectic thickness, scale bar applies on all images.

The eutectic thickness was evaluated from SEM cross-sections for Sn- 20, 30, 35 and 47 wt.% Bi alloys for comparison with 3D measurement. The cross-sections are illustrated in Figure 12, where Figure 12a–d shows the microstructure of the 20, 30 and 47 wt.% Bi alloys, respectively. The white domains in Figure 12a–d show the eutectic phase, embedded in gray primary phase. Secondary precipitates of Bi can be seen in the primary phase, which form due to a reduced solubility of Bi in β-Sn at room temperature. The corresponding local thickness map of the reference samples is illustrated in Figure 13a–d. In case of the Sn-Bi system, the eutectic thickness provides a close estimate of the liquid film above the solidus temperature. The liquid film thickness defines the tensile strength of the alloy in the last stage of solidification [34] and is a determining factor for the formation of solidification cracks. The hot tearing criteria reviewed by Eskin et al. [34] show an inverse relation of liquid film thickness and strength. Therefore, the Sn-20 wt.% Bi alloy is expected to provide the highest mechanical strength in the semi-solid configuration among the studied alloys.

**Figure 14:**
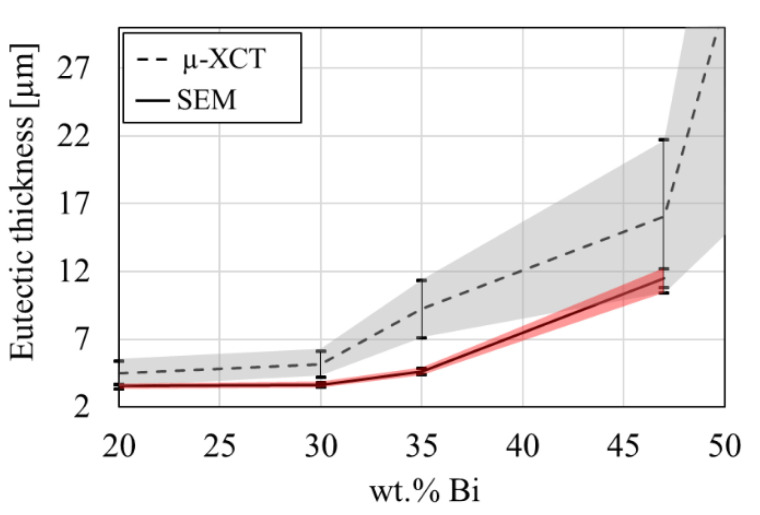
Comparison of the image analyzed eutectic thickness obtained from the µ-XCT data and SEM data as a function of alloy composition. Error bars indicate the standard deviation of eutectic thickness.

**Figure A5:**
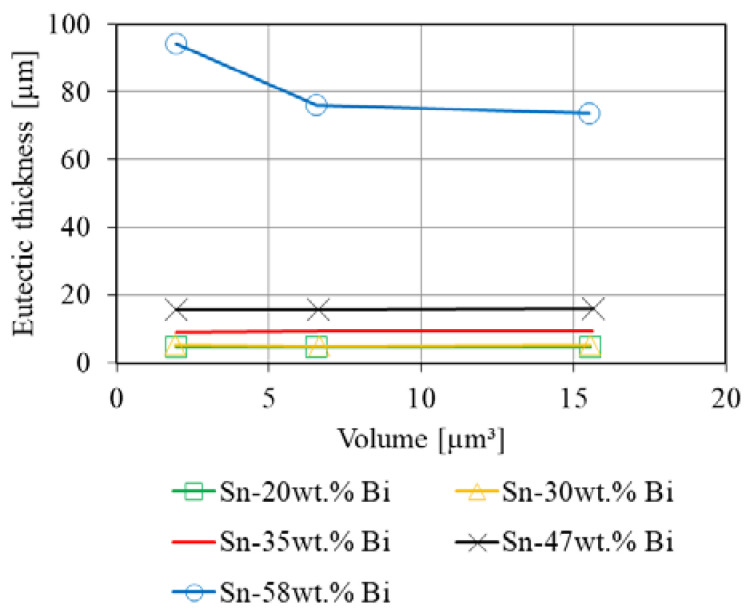
Eutectic thickness over variation of VOI and alloy composition.

**Figure A6:**
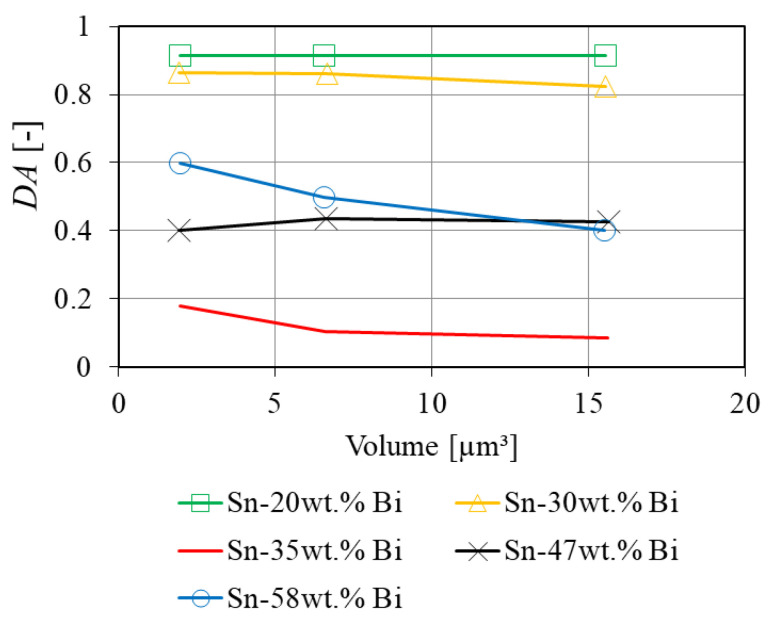
Degree of anisotropy over variation of VOI and alloy composition.

A comparison of µ-XCT and SEM measurements is given in Figure 14. A good correlation between both imaging techniques is observed over alloy compositions. It shows that SEM cross-sections provide a good estimate for the local phase thickness. Nevertheless, 2D measurements give a lower bound estimate for the actual local thickness. This is shown in Figure 14 with SEM measurements being close to lower values of µ-XCT measurements. A possible size-dependence of the eutectic thickness on VOI was investigated with results given in Figure A5.

### 4.3. Morphology Classification

**Figure 15:**
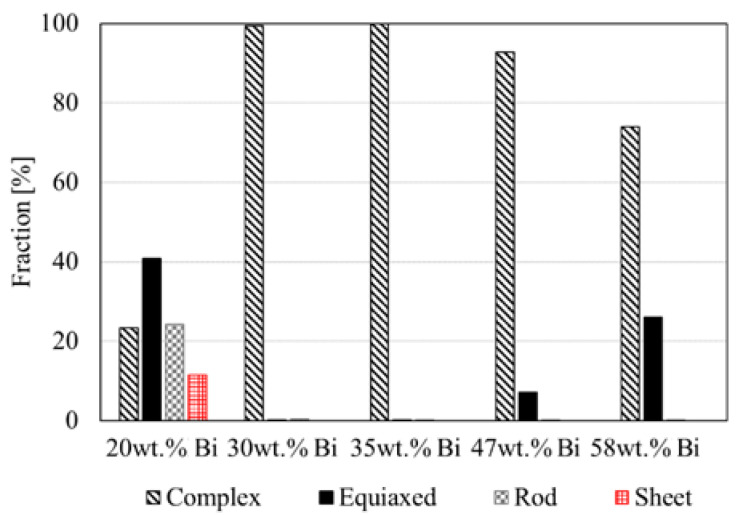
Volume fractions of morphology classes in Sn-Bi alloys obtained from segmented µ-XCT data.

A volumetric classification is given in Figure 15, which shows the volume fraction of each morphologic class for all alloy composition. Detailed values can be found in Appendix D in Table A2. The Sn-20 wt.% Bi alloy is composed of complex, equiaxed, rod and sheet areas, where 40% of the microstructure is composed of equiaxed domains. The Sn-30 wt.% Bi and Sn-35 wt.% Bi alloys reveal complex microstructures due to the large eutectic and primary networks. In Table A2, insignificant fractions of equiaxed and rod-shaped domains are given. The Sn-47 wt.% Bi and Sn-58 wt.% Bi alloys are composed of equiaxed and complex domains, where the equiaxed areas have a minor volume fraction of 7% and 26%. Overall, the relative volume fractions in Figure 15 reveal similar morphologies among some of the investigated alloys. Three categories are suggested to group alloys with similar features. The Sn-20 wt.% Bi alloy represents a “mixed” microstructure with significant amounts of complex, equiaxed, rod and sheet domains. The Sn-30 wt.% Bi and Sn-35 wt.% Bi alloys show a “complex-dominated” structure with negligible contributions of equiaxed- and rod-shaped areas. Furthermore, the Sn-47 wt.% Bi and Sn-58 wt.% Bi alloys are summarized as “complex-equiaxed”, where the microstructure is dominated by complex areas with a significant presence of equiaxed domains. The data in Figure 15 suggests that alloys with eutectic phase fraction between 30 and 60% are complex dominated due to the formation of a fine network of eutectic phase. The dendritic primary phase for alloys with 47 and 58 wt.%Bi leads to a fraction of equiaxed domains of 7% and 26%.

### 4.4. Morphology and Elastic Anisotropy

**Figure 16:**
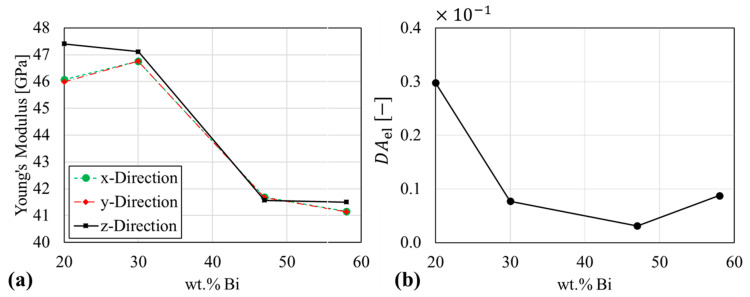
Evaluation of calculated elastic anisotropy from RVE-FEM models (**a**) Young’s moduli from unidirectional tensile deformation (**b**) degree of anisotropy from Young’s modulus.

**Figure A7:**
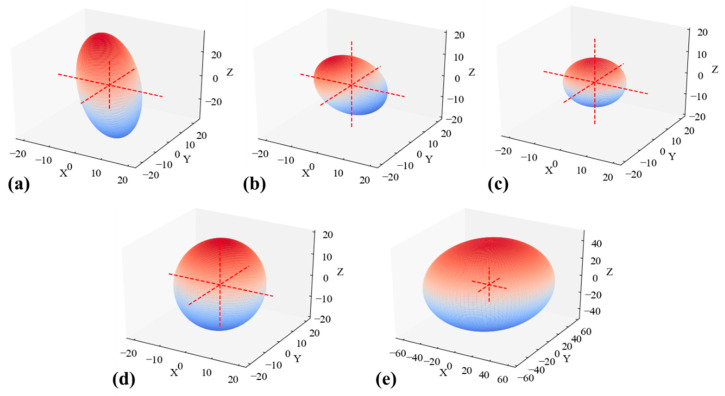
Ellipsoids according to Table A1 of (**a**) Sn-20 wt.% Bi (**b**) Sn-30 wt.% Bi (**c**) Sn-35 wt.% Bi (**d**) Sn-47 wt.% Bi (**e**) Sn-58 wt.% Bi.

To investigate a possible correlation among geometric and elastic anisotropy, Young’s moduli were computed with the RVE-FE models. Figure 16 shows the elastic response over composition, where Figure 16a shows the moduli in each spatial direction of the unit cell. Figure 16b shows the elastic $DA_{\mathrm{el}}$ computed according to Equation (5). The Sn-20 wt.% Bi alloy shows the most pronounced orientation dependency, with a 1.4 GPa higher modulus in z-direction. This agrees with the equivalent ellipsoid stretched in z-direction in Figure A7. The remaining alloys show a negligible orientation-dependence. The elastic $DA_{\mathrm{el}}$ parameter in Figure 16b indicates an isotropic characteristic for all alloys. The highest anisotropy is found for the Sn-20 wt.% Bi alloy with $DA_{\mathrm{el}}=0.03$. The similar elastic constants of primary and eutectic phase reduce the geometric anisotropy found in µ-XCT Data shown in Figure 9. Furthermore, it is important to discuss the effect of crystal anisotropy on the elastic properties. In principle, single grains or grain boundaries are hardly detected through lab-scale µ-XCT. Nevertheless, a similar approach was reported by Wijaya et al. [51], where the local crystal orientation of the microstructure was neglected and good correlation of experimental and numerical results of elastic properties was achieved. However, in case of solder joints, the local crystal anisotropy becomes a determining factor for solder joint reliability [52]. The body-centered tetragonal crystal structure causes an elastic and thermal expansion misfit, where large-angle grain boundaries act as damage initiation sites.

### 4.5. Semi-Solid Shear Strength

The microstructural and morphology descriptors reported in the present work allow a discussion of several mechanical properties in the semi-solid state, with the eutectic phase still being liquid [35,36]. Under these conditions, the residual strength of the solder joint is an important measure to estimate tolerable external forces. The shear strength of Sn-Pb semi-solids of varying microstructure was studied by Martin et al. [53], which is used for a comparative discussion due to its close similarity to the Sn-Bi system. The authors reported a morphology-dependence of shear strength, where equiaxed microstructures showed lower values of about 1/10 compared to those for dendritic microstructures. The liquid fraction also influenced the shear strength in the semi-solid state, with values ranging from 3 MPa at liquid fraction of $\phi_{L}=20\%$ to 1.2 MPa at $\phi_{L}=37\%$. Assuming a condition close to the solidus temperature of $T_{S}=138\;{}^{\circ}C$. the liquid fraction of Sn-Bi alloys is equal to the volume fraction of eutectic, where measured values are given in Table 3. Therefore, one can conclude that the expected shear strength during healing of the Sn-20 wt.% Bi alloy will be >3 MPa, as a result of the presences of the solid skeleton of β-Sn illustrated in Figure 4a. The remaining alloys with 30 wt.% to 58 wt.% Bi are likely to have a shear strength <1 MPa in comparison with [53]. The shear strengthening effect of dendritic microstructures reported by Martin et al. [53] in case of the Sn-58 wt.% Bi is most likely overcompensated by the high liquid fraction of $\phi_{L}=94.7\%$. Furthermore, the presence of a liquid film network shown in Figure 3b–d is an additional limitation of semi-solid strength. The above analysis provides an estimate for choosing the handling conditions of solder joints during liquid-assisted healing. 

### 4.6. Semi-Solid Uniaxial Compression

Theoretical considerations of liquid-assisted healing reported in References. [35,36] suggest that external compressive deformation is a driving force for liquid-assisted healing. This effect is limited by the tendency of void formation under semi-solid compression, which has been investigated for Al-Cu alloys [54,55,56]. The eutectic character of this alloy system offers enough similarity with Sn-Bi solders for qualitative comparison. Kareh et al. [55] studied void formation in globular-equiaxed microstructures under variation of liquid fraction. The alloys deformed similarly to a cohesion-less granular material and showed a varying tendency of void formation under uniaxial compression, which was associated with shear-induced dilation of the microstructure. A critical liquid fraction of $\phi_{S}=37.6\%$ was found above which void formation was fully inhibited. A minimal liquid channel thickness is required to compensate dilation and accommodate grain movement. In case of interacting solid grains, which is likely when the liquid film thickness drops below the minimal required value, void formation is observed. These results suggest that Sn-Bi alloys with >30 wt.% Bi provide the required liquid film thickness to be insensitive to void formation during compression. The work of Cai et al. [56] supports this suggestions, as void shrinkage under compressive strains of ϵ<1.2% in the semi-solid was observed in alloys with $\phi_{S}=30\%$. Further uniaxial compression, however, leads to nucleation and growth of voids as soon as the liquid film thickness shrinks, inducing local tensile stresses and inhibiting liquid feeding. The microstructural data of Sn-Bi alloys, therefore, suggest a varying degree of maximum compression to be applied until void nucleation appears in the liquid. The critical value of $\phi_{S}$ reported by Kareh et al. [55] is fulfilled for Sn alloys with 35, 47 and 58 wt.% Bi. It is theorized that the Sn-35 wt.% Bi alloy with an average liquid film thickness of 9.2 μm will tolerate less uniaxial compression than the Sn- 47 wt.% and Sn- 58 wt.% Bi alloys with 16 μm and 73 μm liquid thickness, respectively, due to early closing of the liquid film. Further semi-solid compression experiments on these alloys could reveal the degree of tolerable deformation and provide a link between microstructural features and semi-solid void formation. In particular, the evolution of morphology parameters, such as, $\Omega_{3},\;E,\;F$ with respect to semi-solid deformation, can identify the ‘safe’ and the ‘damage-creating’ conditions.

### 4.7. Semi-Solid Permeability

**Figure 17:**
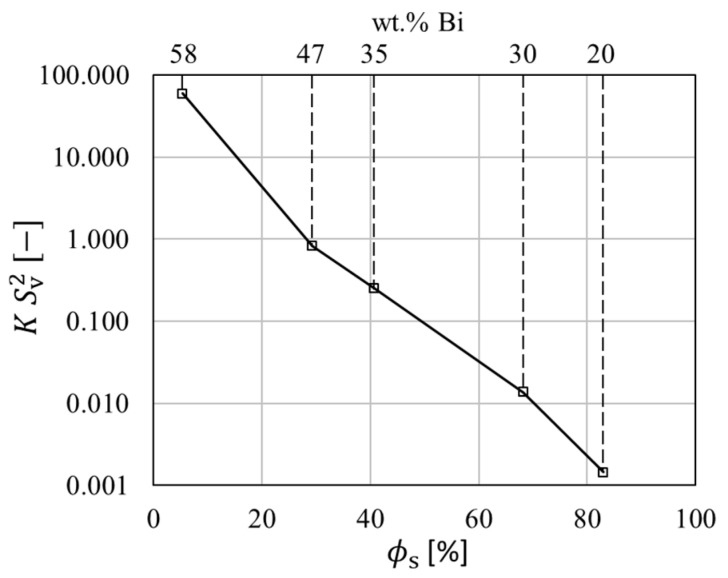
Dimensionless permeability over solid fraction (just before the final formation of the eutectic fraction) and Sn-Bi alloy composition, calculated from µ-XCT measurements.

The theoretical considerations of liquid-assisted healing in References. [35,36] discussed the role of local material transport for void healing. It was mentioned that the local resistance against material transport will lead to a deviation of the theoretical healing efficiency. The Carman-Kozeny relation [57] is used to describe the permeability, *K*. This quantity provides an estimate for resistance against liquid flow as

(10) $$K=\frac{{\left(1-f_{s}\right)}^{3}}{k_{c}S_{v}^{2}f_{s}^{2}}$$

with $f_{s}$ being the solid fraction, $k_{c}$ being a dimensional constant (3 for dendritic and 5 for equiaxed structures) and $S_{v}$ the surface fraction. The dimensionless permeability indicates the required pressure gradient for fluid flow and is given in Figure 17 from measured values of $f_{s}$ and $S_{v}$ according to Equation (9). The pressure gradient and fluid flow was investigated by 5.

### 4.8. Implications for Liquid Phase Healing

The discussion of shear strength, uniaxial compression and permeability in the semi-solid state provides necessary criteria for liquid phase healing. The presence of a liquid phase network is required to increase the microstructural permeability and to allow material transport for defect filling, which is fulfilled for the investigated solders except the Sn-20 wt.% Bi alloy. Furthermore, alloys with 35, 47 and 58 wt.% Bi provide a liquid fraction larger than the critical value of 38% reported by Kareh et al. [55] to prevent formation of porosities during compression. These alloys require a load free condition during healing, due to the low expected shear strength in the semi-solid state. Therefore, solders of 35, 47 and 58 wt.% Bi show the highest potential of liquid phase healing in absence of external loads.

## 5. Conclusions

This work presents a thorough microstructural analysis of µ-XCT data of five Sn alloys with varying Bi content. The following outcomes are reported:

1. Eutectic phase fractions measured with µ-XCT showed good correlation with expected values from CALPHAD calculations.
2. The Sn-20 wt.% Bi alloy show fragmented domains of eutectic embedded in primary phase. The Sn-35 wt.% Bi alloy reveals interpenetrating primary and eutectic phase, which is reflected by the highest phase boundary area fraction. The Sn-58 wt.% Bi, despite its high eutectic phase fraction, showed a connected dendritic network of primary phase.
3. The morphology analysis shows microstructural similarities of the investigated alloys. The Sn-20 wt.% Bi alloy is composed of four geometric features and its microstructure is categorized as “mixed”. The alloys with 30 wt.% and 35 wt.% Bi are composed of mainly “complex-shaped” areas. The alloys of near-eutectic composition, Sn- 47 wt.% Bi and 58 wt.% Bi, constitute from “complex” and “equiaxed” domains.
4. The RVE-FE simulations showed an elastically isotropic response, despite significant spatial orientation of the microstructures. The similar elastic properties of primary and eutectic phase reduce the impact of geometric anisotropy. Results are in agreement with linear and inverse rule of mixture.

Finally, it can be concluded that the analysis of liquid phase fraction, liquid film thickness and permeability suggests that among the investigated Sn-Bi alloys, those with a Bi content between 35 and 58 wt.% have the highest potential for liquid phase healing. The low mechanical strength in this configuration requires a load free condition during healing.

## Figures and Tables

**Table 1:** Values for shape categorization based on complexity parameter $\Omega_{3}$, elongation *E* and flatness *F* [39].

	*E*	*F*	$\Omega_{3}$
**Sphere**	-	-	$\Omega_{3}$ ≤ 1.15
**Equiaxed**	<5	<5	1.15 < $\Omega_{3}$ ≤ 2.5
**Rod**	≥5	<5	1.15 < $\Omega_{3}$ ≤ 2.5
**Sheet**	-	≥5	1.15 < $\Omega_{3}$ ≤ 2.5
**Complex**	-	-	$\Omega_{3}$ ≥ 2.5

**Table 2:** Elastic material parameters of RVE-FE model [17].

Property	Value [Unit]
Young’s modulus primary phase	50.0 [GPa]
Poisson number primary phase	0.33 [-]
Young’s modulus eutectic phase	40.0 [GPa]
Poisson number eutectic phase	0.33 [-]

**Table 3:** Liquid fraction at solidus temperature over solder composition.

Composition	$\phi_{L}$ $\;\mathrm{at}\;T_{S}$
Sn-20 wt.% Bi	17.1%
Sn-30 wt.% Bi	31.8%
Sn-35 wt.% Bi	59.4%
Sn-47 wt.% Bi	70.8%
Sn-58 wt.% Bi	94.7%

## Data Availability

Data sharing is not applicable to this article.

## Appendix C. Ellipsoid Fit

**Table A1:** Degree of anisotropy, eigenvectors $m_{ij}$ and eigenvalues $\lambda_{i}$ of ellipsoid fit.

wt.% Bi	DA	$m_{11}$	$m_{12}$	$m_{13}$	$m_{21}$	$m_{22}$	$m_{23}$	$m_{31}$	$m_{32}$	$m_{33}$	$\lambda_{1}$	$\lambda_{2}$	$\lambda_{3}$
20	0.914	0.159	−0.119	−0.980	0.415	−0.893	0.176	0.896	0.434	0.093	0.0008	0.0018	0.0088
30	0.825	−0.317	−0.776	0.545	−0.339	0.629	0.700	0.886	−0.037	0.463	0.0036	0.0067	0.0208
35	0.085	0.690	0.676	−0.259	0.660	0.734	−0.160	−0.299	−0.061	0.952	0.0089	0.0090	0.0097
47	0.425	0.061	0.059	0.996	0.808	0.589	−0.014	0.586	0.806	0.083	0.0025	0.0038	0.0044
58	0.401	0.824	0.565	0.050	0.564	−0.825	0.032	0.059	0.002	−0.998	0.0002	0.0004	0.0004

## Appendix D. Volume Fractions of Morphologic Classes

**Table A2:** Volume fraction of morphologic classes.

wt.% Bi	Equiaxed	Rod	Sheet	Complex
58	26.034	0.033	0.0	74.0
47	7.076	0.108	0.0	92.8
35	0.065	0.002	0.0	99.9
30	0.244	0.271	0.0	99.5
20	40.859	24.171	11.5	23.4
